# Mechanobiological regulation of placental trophoblast fusion and function through extracellular matrix rigidity

**DOI:** 10.1038/s41598-020-62659-8

**Published:** 2020-04-03

**Authors:** Zhenwei Ma, Lucas Sagrillo-Fagundes, Stephanie Mok, Cathy Vaillancourt, Christopher Moraes

**Affiliations:** 1 Department of Chemical Engineering, McGill University, Montréal, QC Canada; 2 INRS-Centre Armand Frappier Santé Biotechnologie and Réseau Intersectoriel de Recherche en Santé de l’Université du Québec, Laval, QC Canada; 3 Center for Interdisciplinary Research on Well-Being, Health, Society and Environment, Université du Québec à Montréal, Montréal, QC Canada; 4 Department of Biological and Biomedical Engineering, McGill University, Montréal, QC Canada; 5 Rosalind and Morris Goodman Cancer Research Centre, McGill University, Montréal, QC Canada

**Keywords:** Mechanisms of disease, Tissues, Tissue engineering

## Abstract

The syncytiotrophoblast is a multinucleated layer that plays a critical role in regulating functions of the human placenta during pregnancy. Maintaining the syncytiotrophoblast layer relies on ongoing fusion of mononuclear cytotrophoblasts throughout pregnancy, and errors in this fusion process are associated with complications such as preeclampsia. While biochemical factors are known to drive fusion, the role of disease-specific extracellular biophysical cues remains undefined. Since substrate mechanics play a crucial role in several diseases, and preeclampsia is associated with placental stiffening, we hypothesize that trophoblast fusion is mechanically regulated by substrate stiffness. We developed stiffness-tunable polyacrylamide substrate formulations that match the linear elasticity of placental tissue in normal and disease conditions, and evaluated trophoblast morphology, fusion, and function on these surfaces. Our results demonstrate that morphology, fusion, and hormone release is mechanically-regulated via myosin-II; optimal on substrates that match healthy placental tissue stiffness; and dysregulated on disease-like and supraphysiologically-stiff substrates. We further demonstrate that stiff regions in heterogeneous substrates provide dominant physical cues that inhibit fusion, suggesting that even focal tissue stiffening limits widespread trophoblast fusion and tissue function. These results confirm that mechanical microenvironmental cues influence fusion in the placenta, provide critical information needed to engineer better *in vitro* models for placental disease, and may ultimately be used to develop novel mechanically-mediated therapeutic strategies to resolve fusion-related disorders during pregnancy.

## Introduction

The human placental barrier is responsible for several critical functions during pregnancy including nutrient transport, gas exchange, waste elimination and hormone secretion^1^. The placenta hence directly impacts fetal development^2^, immune tolerance^3^, and gestational length^4^, each of which can profoundly affect long-term quality of life and healthcare economics for both mother and baby^5–8^. Transport across this fetal-maternal interface is regulated by the syncytiotrophoblast, a multinucleated layer that forms the outer surface of the placental villi^9^. The syncytiotrophoblast arises and is maintained by continuous fusion of mononuclear villous cytotrophoblasts (vCTBs)^10^, through a tightly regulated process that can only be partially recreated *in vitro*^11^. Disruption of fusion results in placental malformation and aberrant villous trophoblast turnover^10^, which is associated with life-altering pregnancy complications such as preeclampsia^12^ and intrauterine growth restriction^13^.

Several biochemical factors are known to regulate placental trophoblast fusion *in vitro* and *in vivo*, including growth factors^14–16^, hormones^17^, proteases^18–20^, transcription factors^21^ and membrane proteins^22^. Despite this wealth of information, fusion remains a stochastic and poorly controlled process in cultured cells, making it challenging to establish *in vitro* models of placental function. These difficulties suggest that other non-biochemical factors could play a significant and previously under-appreciated role in the trophoblast fusion process. Understanding how microenvironmental cues might influence fusion, particularly for those factors associated with diseases, could hence have a significant impact on our capacity to construct models for fundamental studies of fusion, develop strategies to modulate the fusion process, and ultimately resolve fusion-related disorders during pregnancy.

**Figure 1:**
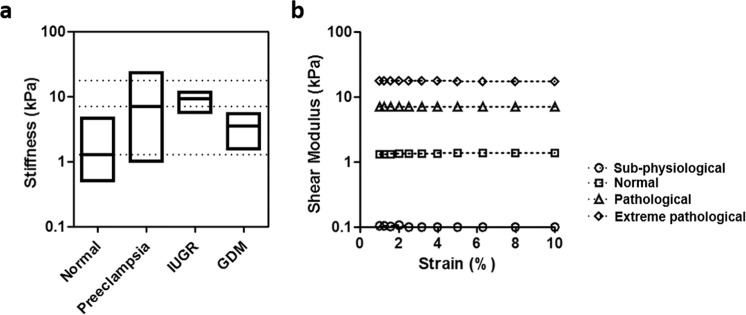
Design criteria and mechanical properties for stiffness-tunable substrates. (**a**) Tissue stiffness of normal and diseased placenta (log scale, shear modulus values), using data obtained from literature^26–30^. (**b**) Measured shear modulus of synthesized polyacrylamide hydrogels measured over an applied strain of 10%. Loss modulus measurements were negligible.

Tissue stiffness is now well-established as a critically important regulator of a wide variety of cellular processes, including differentiation^23^ and disease progression^24^; but the role of extracellular tissue mechanics on trophoblast fusion has not previously been defined. Recent studies have demonstrated that ECM thickness affects fusion-related markers of mRNA and secreted proteins in trophoblasts^25^ suggesting that mechanics may play a role, but whether trophoblast fusion is mechanically sensitive to disease-specific physiological cues remains undefined. Interestingly, recent evidence from *in vivo* imaging studies demonstrates that tissue stiffness varies significantly across patients diagnosed with pregnancy complications, including preeclampsia^26–28^, intra-uterine growth restriction^29^, and gestational diabetes mellitus^30^ compared to normal placental tissue^26^ (literature values presented in Fig. 1a). The general trend towards increased tissue stiffness in disease conditions further suggests that tissue mechanics affect fusion efficiency, and ultimately syncytiotrophoblast function.

Here, we hypothesize that disease-relevant substrate tissue mechanics are regulatory factors for trophoblast fusion, and we test this idea using a stiffness-tunable hydrogel culture system. In contrast with standard culture on supra-physiologically stiff plastic or glass substrates^31^, hydrogel composition can be altered to recapitulate the range of mechanical rigidity measured in normal and pathological placental tissue. Using a human placental choriocarcinoma line frequently used for fusion studies, and primary vCTBs isolated from human placenta at term, we test the effects of substrate stiffness and focal sites of elevated stiffness^32,33^ on trophoblast morphology, fusion efficiency, and secretory functions.

## Results

### Mechanical characterization of stiffness-tunable substrates

Stiffness-tunable polyacrylamide hydrogels were selected for these studies, as polyacrylamide composition can be altered to span the physiological range of linear elastic mechanical properties, maintains mechanical properties even in prolonged culture, and can be functionalized with a wide variety of surface adhesive proteins^34^. Here, polyacrylamide hydrogels were fabricated on glass coverslips with varying monomer and crosslinker content based on published protocols^35^. Polyacrylamide surfaces were functionalized with type I collagen (data not shown), an abundantly available ECM protein in placental tissue^36^. Shear rheometry was used to characterize the mechanics of polymerized hydrogels (Fig. 1b), and demonstrated stable, linear elastic mechanical properties up to 10% strain for all hydrogel formulations tested. Loss modulus measurements from these substrates were negligible. For the studies presented here, polyacrylamide hydrogel formulations representing sub-physiological (shear modulus G = 0.1 kPa), normal (G = 1.3 kPa), pathological (G = 7 kPa; preeclamptic), and extreme pathological (G = 17.4 kPa; preeclamptic^26^) mechanical rigidities were selected, and compared with culture on standard glass substrates (~10^6^ kPa).

### Substrate stiffness regulates trophoblast cell morphology

Cell morphology is intimately linked with cell function, through rearrangements in the internal cytoskeletal structure, and in response to external mechanical culture conditions^37–39^. To characterize cell morphology on substrates of controlled stiffness, human placental choriocarcinoma cells (BeWo) commonly used for studies of fusion^31^ were seeded on the functionalized polyacrylamide hydrogel surfaces, and we evaluated morphology and cytoskeletal architecture in relation to substrate stiffness.

**Figure 2:**
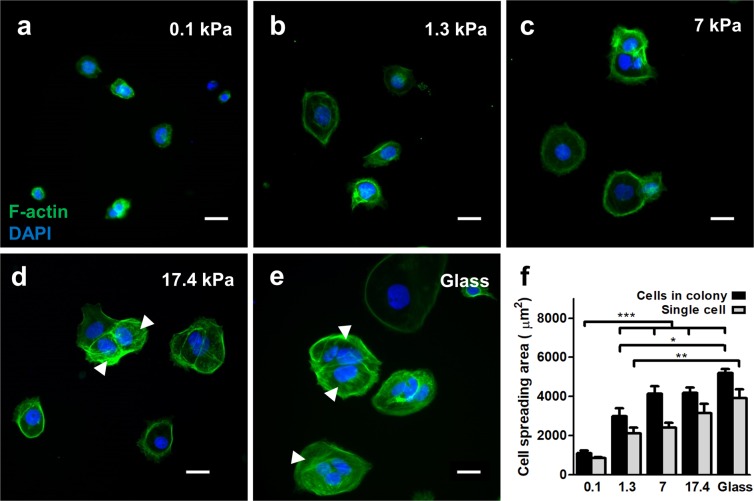
Effects of substrate stiffness on BeWo cell morphology. (**a**–**e**) Representative figures of BeWo cells cultured on substrates with (**a**) 0.1 kPa, (**b**) 1.3 kPa, (**c**) 7 kPa, (**d**) 17.4 kPa, and (**e**) glass substrates. Green: f-actin; blue: nuclear DAPI. Arrows indicate stress fibers. Scale bar is 50 μm. (**f**) Measured average cell spread area of BeWo cells cultured on various substrates. (data reported as mean ± standard deviation for n = 3 independent experiments; *p < 0.05; **p < 0.01; ***p < 0.001, by two-tailed, one-way ANOVA with Holm-Sidak Post-hoc comparison).

Consistent with other adherent cells^35^, BeWo cells adhered and grew as either single cells or small colonies and proliferated rapidly on stiffer surfaces (data not shown). To prevent capturing the cell-cycle dependent effects on cell morphology, proliferation was inhibited on all substrates with mitomycin-C (10 μg/ml). Within 48 hours of culture, cells adopted near-spherical structures on sub-physiologically soft substrates and flat, well-spread morphologies on normal and pathologically stiff substrates (Fig. 2), consistent with many other adherent cell types^35,40^. Also as expected, projected nuclear area decreased with spread area (data not shown). This effect was observed in both single cells and in the average cell area within small colonies (Fig. 2f). Spread area of single cells and cells in colonies on 0.1 kPa gels were significantly lower than all other conditions tested; and significantly greater on glass substrates compared to hydrogels with normal placental stiffness. Hence, cell morphology is sensitive to substrate mechanics, and, if placental fusion is mechanically sensitive, conventional glass or plastic culture substrates would not capture these effects.

We also observed distinct cytoskeletal structures in cells based on the stiffness of the underlying substrate. For all cells, the cytoskeleton displayed cortical filamentous actin structures at the cell boundaries for all substrates (Fig. 2a–e). When cell-cell contact was made, clearly distinct f-actin stress fibers were observed spanning the multicellular structure, and were qualitatively more abundant in cells cultured on substrates with higher stiffness. These qualitative observations suggest increased intercellular mechanical tension^38^.

Interestingly, isolated single cells exhibited greater spreading compared to cells in colonies (Fig. 2f), indicating that neighboring cells limit spreading and hence internal tension to some degree^41^. Since contact with neighboring cells simultaneously increases mechanical tension through stress fiber formation, this would suggest that stresses on individual cells within a monolayer would vary considerably depending on the mechanical activity of neighbouring cells, and that this effect would be biased based on extracellular tissue stiffness^42^. Taken together, these results demonstrate that cell morphology and cytoskeletal activity in trophoblast cells are non-deterministically biased by stiffness of the extracellular matrix, suggesting that any cellular processes linked to these structures would also be affected.

### Substrate stiffness modulates fusion through myosin-II activity

**Figure 3:**
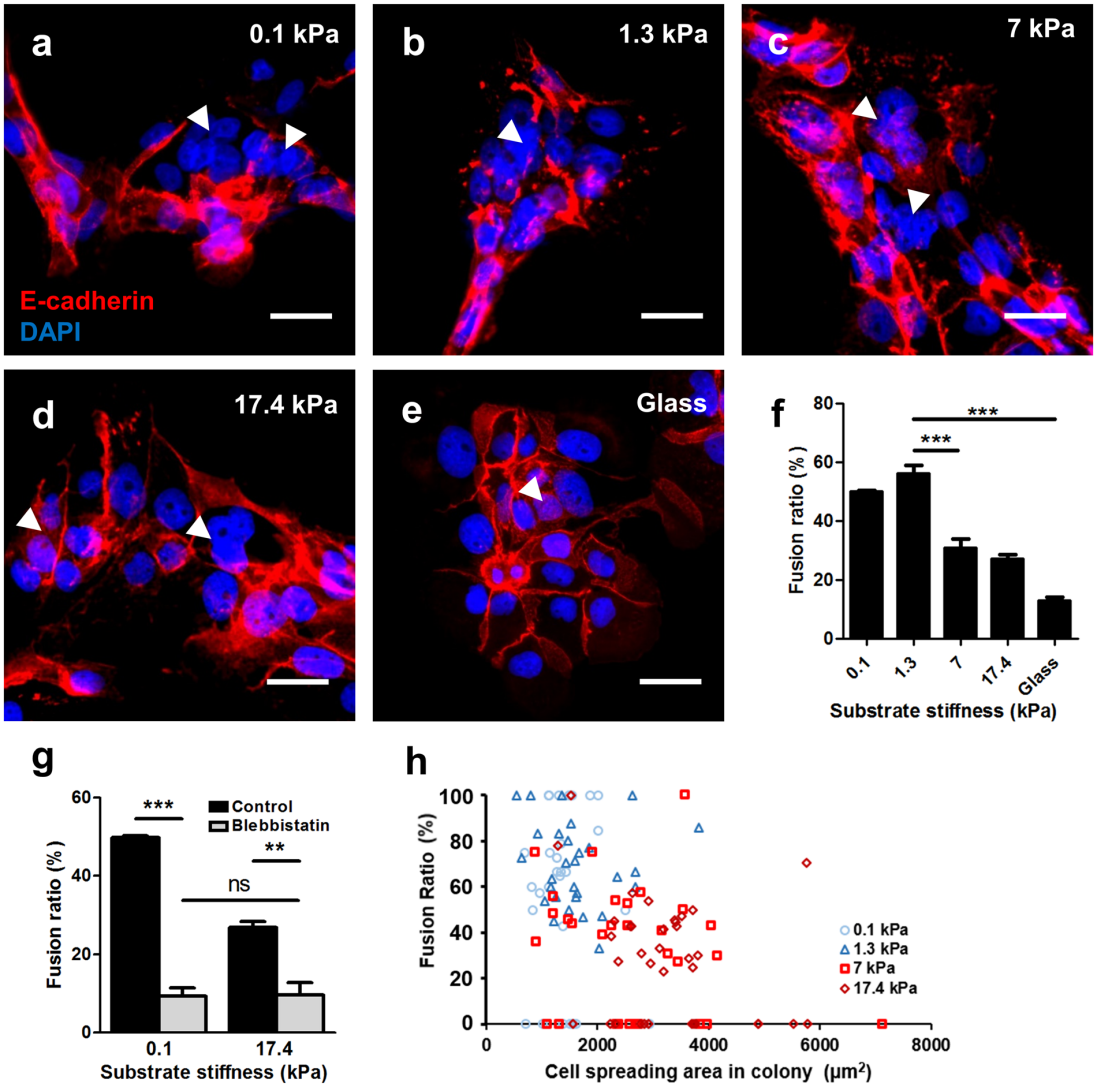
Effects of substrate stiffness on BeWo cell fusion. (**a**–**e**) Representative figures of syncytium formed on substrates with (**a**) 0.1 kPa, (**b**) 1.3 kPa, (**c**) 7 kPa, (**d**) 17.4 kPa, and (**e**) glass substrates. Red: E-cadherin; blue: DAPI. Arrowheads indicate syncytial regions. Scale bar is 50 μm. (**f**) BeWo cell fusion ratio was greatly enhanced on substrate matching normal placental tissue stiffness. (**g**) Fusion was greatly suppressed after blebbistatin inhibition for cells cultured on ultrasoft and ultrastiff substrates. Data reported as mean ± standard deviation for n = 3 independent experiments; **p < 0.01, ***p < 0.001, by Student’s *t*-test. (**h**) No correla*t*ion was found between cell spreading area and fusion ratio for cells cultured on all the substrate stiffness tested.

Fusion in BeWo cells can be induced by forskolin^31^, and evaluated using several methods, the most definitive of which is disruption of the E-cadherin rich plasma membrane between adjacent nuclei in a fused syncytium^43^. The fusion efficiency reported for BeWo cells cultured on glass or plastic is established to be variable, and is sensitive to cell history^44,45^, forskolin activity, and induction time^46^. In our hands, fusion was observed after 48 hours of induction with 20 µM forskolin on all substrates tested (Fig. 3a–e). In addition to E-cadherin disruption, nuclei were observed to aggregate within the syncytial patches, as expected during fusion^43^. Following standard metrics to quantify fusion in these cultures^43^, we demonstrate that fusion is significantly affected by substrate stiffness (Fig. 3f), and was four-fold greater (~60% compared ~15%) on softer substrates that mimic normal placental tissue stiffness (1.3 kPa) than on glass. In contrast, fusion efficiency on substrates that mimic pathological and extreme pathological stiffnesses (>7 kPa) were not significantly different from culture on glass.

Polyacrylamide hydrogels of differential stiffness have also been reported to vary in porosity, and micro/nano-scale architecture^47^, which may influence interpretation of these results. To confirm that the substrate stiffness effects on fusion efficiency are mechanically regulated, cells were treated with blebbistatin, a myosin II inhibitor, during the 48 hours of culture in fusion induction media. Cell fusion ratios were significantly suppressed on both soft and stiff tissues when treated with blebbistatin (Fig. 3g), confirming that this effect is mechanically mediated through myosin-II activity. Low levels of fusion persisted, similar to those observed on glass substrates (~10–15%). Since blebbistatin reduces internal cytoskeletal tension, and culture on glass increases tension, these results provide further evidence that an optimal internal stress level or pattern exists to promote fusion.

Since some variations in cell spread area do exist within cell colonies on substrates of varying stiffness (Fig. 2f), we then asked whether fusion was correlated with spread area of cells within individual colonies. Hence, fusion ratios were analyzed based on average cell spread area on a per-colony basis (Fig. 3h). No correlative trend was observed between cell size and fusion efficiency, indicating that stiffness-modulated fusion occurs independently from cell spread area.

### Substrate stiffness modulates syncytial secretory functions

Human chorionic gonadotropin (hCG) is a hormone produced by the placenta which plays a critical role in establishing and maintaining normal pregnancy, and is considered an important functional biochemical marker of trophoblast differentiation^17^. However, excessive hCG production is also a marker of disease progression in placental disorders such as gestational trophoblastic disease and preeclampsia^48–51^, and elevated hCG is thought to reflect early placental damage or dysfunction^52^. Hence, an optimal hCG secretion level likely exists for fused cells, and here we quantified both intracellular (immunofluorescent staining) and released levels (ELISA) of a beta subunit of hCG as a function of substrate stiffness.

**Figure 4:**
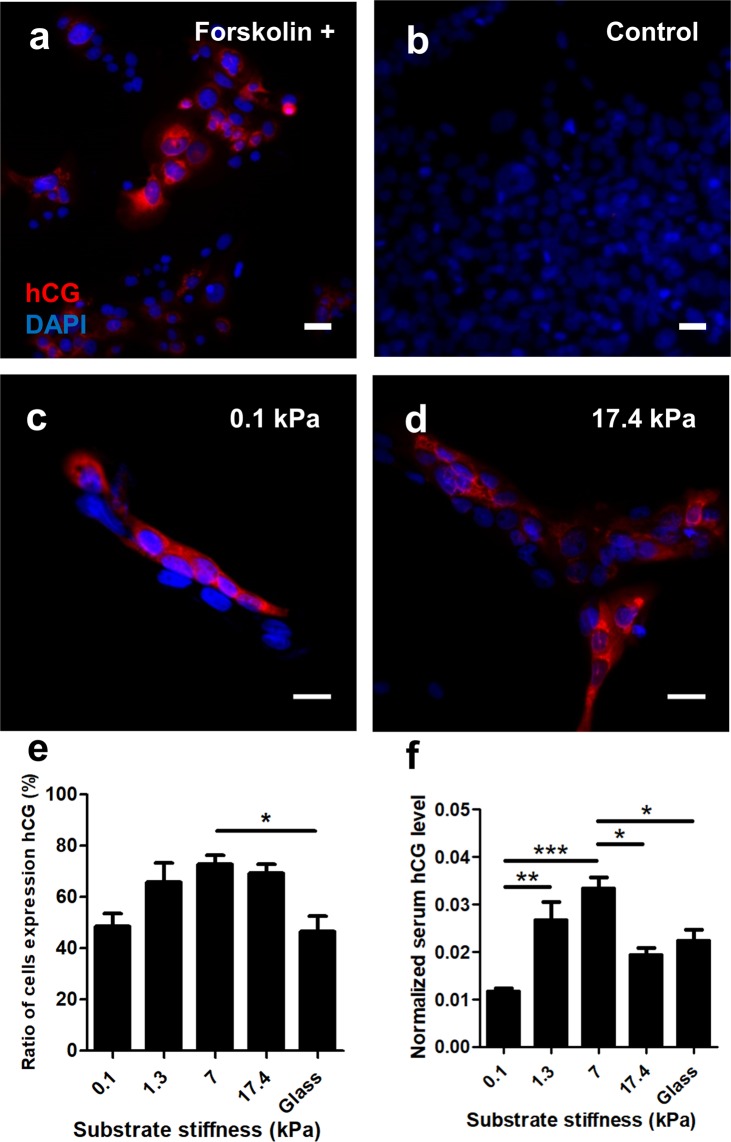
Effects of substrate stiffness on BeWo cell β-hCG expression. (**a**,**b**) BeWo cells only express β-hCG with forskolin treatment. (**c**,**d**) Representative figures of cells expressing β-hCG on soft (**c**) and stiff (**d**) substrates. Red: β-hCG; blue: DAPI. Scale bar is 50 μm. (**e**) Ratio of BeWo cells expressing β-hCG and (**f**) normalized media β-hCG level when cultured on substrates with various stiffness. Data reported as mean ± standard deviation for n = 3 independent experiments; *p < 0.05, **p < 0.01, ***p < 0.001, by two-tailed, one-way ANOVA with Holm-Sidak Post-hoc comparison.

We first verified that forskolin induction is required for β-hCG production (Fig. 4a,b). The fraction of β-hCG positive cells depended on substrate stiffness, with only half the cells on the softest substrates producing detectable β-hCG, despite a high fusion efficiency (Fig. 4c–e). In contrast, a large fraction of cells on disease-like substrates were β-hCG positive (Fig. 4e), despite having low fusion efficiency. Analysis of released β-hCG further confirmed this trend, demonstrating that released β-hCG levels are altered based on substrate stiffness (Fig. 4f). In contrast, the released β-hCG levels dropped for cells cultured on supraphysiologically stiff hydrogels and on glass substrates, suggesting a highly complex regulatory mechanism for β-hCG production, and highlighting the need for further studies of fusion processes on hydrogels with physiologically-realistic mechanical properties. Together, these results do demonstrate that functional secretion of β-hCG is substrate-stiffness dependent, suggesting a novel mechanism that impacts production of this important regulatory hormone.

### Effects of substrate stiffness on primary isolated trophoblasts

**Figure 5:**
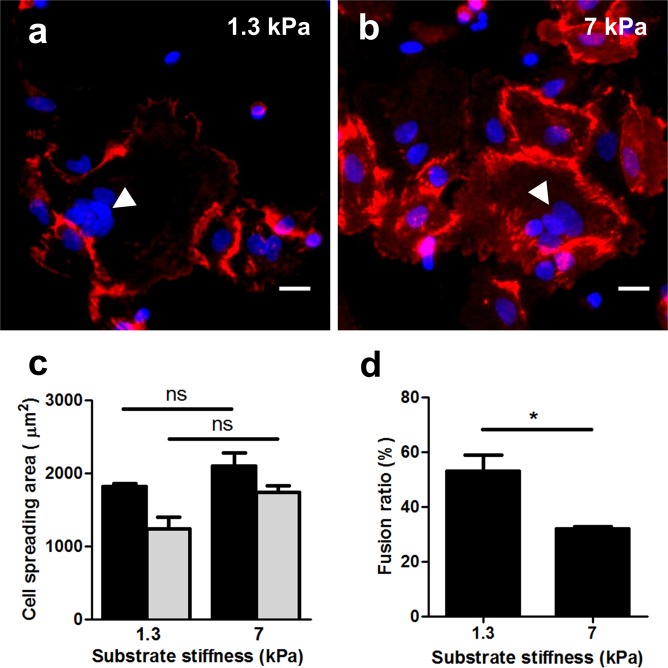
Effects of substrate stiffness on vCTBs morphology and fusion. (**a**,**b**) Representative figures of vCTBs cultured on substrates with normal (**a**) and preeclamptic (**b**) tissue stiffness after 72-hour primary culture. Red: E-cadherin; blue: DAPI. Scale bar is 50 μm. (**c**) No significant difference was observed for vCTBs cultured on different substrates stiffness tested. (**d**) Fusion ratio was greatly enhanced for vCTBs cultured on substrate matching normal placental tissue stiffness. Data reported as mean ± standard deviation for n = 3 independent experiments; *p < 0.05, by Student’s *t*-test.

To verify that the observed effects are not an artifact of using a cell line, we conducted limited additional studies on placental vCTBs isolated from patients at the time of natural vaginal delivery. These cells exhibit distinct characteristics compared to BeWo cells, making them challenging to work with: they do not proliferate *in vitro*, limiting the availability of the cells; and in our hands they spontaneously fuse into syncytial patches within 72 hours without forskolin induction. vCTBs all adopted a similar flat morphology and tended to form a giant cell-sheet structure (Fig. 5a,b) on both normal and preeclamptically stiff substrates. Unlike BeWo cells, no significant difference was observed in cell spread area in these two conditions (Fig. 5c). However, the fusion ratio of vCTBs cultured on substrates with normal tissue stiffness (1.3 kPa) showed a nearly two-fold increase in fusion compared to those cultured on preeclamptically stiff substrates (7 kPa; Fig. 5d), confirming that substrate stiffness plays a critical role in fusion in near-native primary cells, as well as in cell lines.

### Effects of heterogeneous substrate stiffness on trophoblast fusion

Our results demonstrate that trophoblast fusion and function is mechanically sensitive and directly regulated by substrate stiffness on substrates with homogenous rigidity. *In vivo* tissue rigidity is significantly more heterogenous, as tissue stiffening is typically a highly focal process, driven by calcification^53^, inflammation^54^ or cell remodeling activity^55^ that tends to occur in localized regions. This is reflected in *in vivo* imaging data^26^ which shows large heterogeneity within the same tissue^26^. To begin addressing this question, we asked whether fusion events would be spatially biased on substrates with an integrated pattern of varying stiffness.

**Figure 6:**
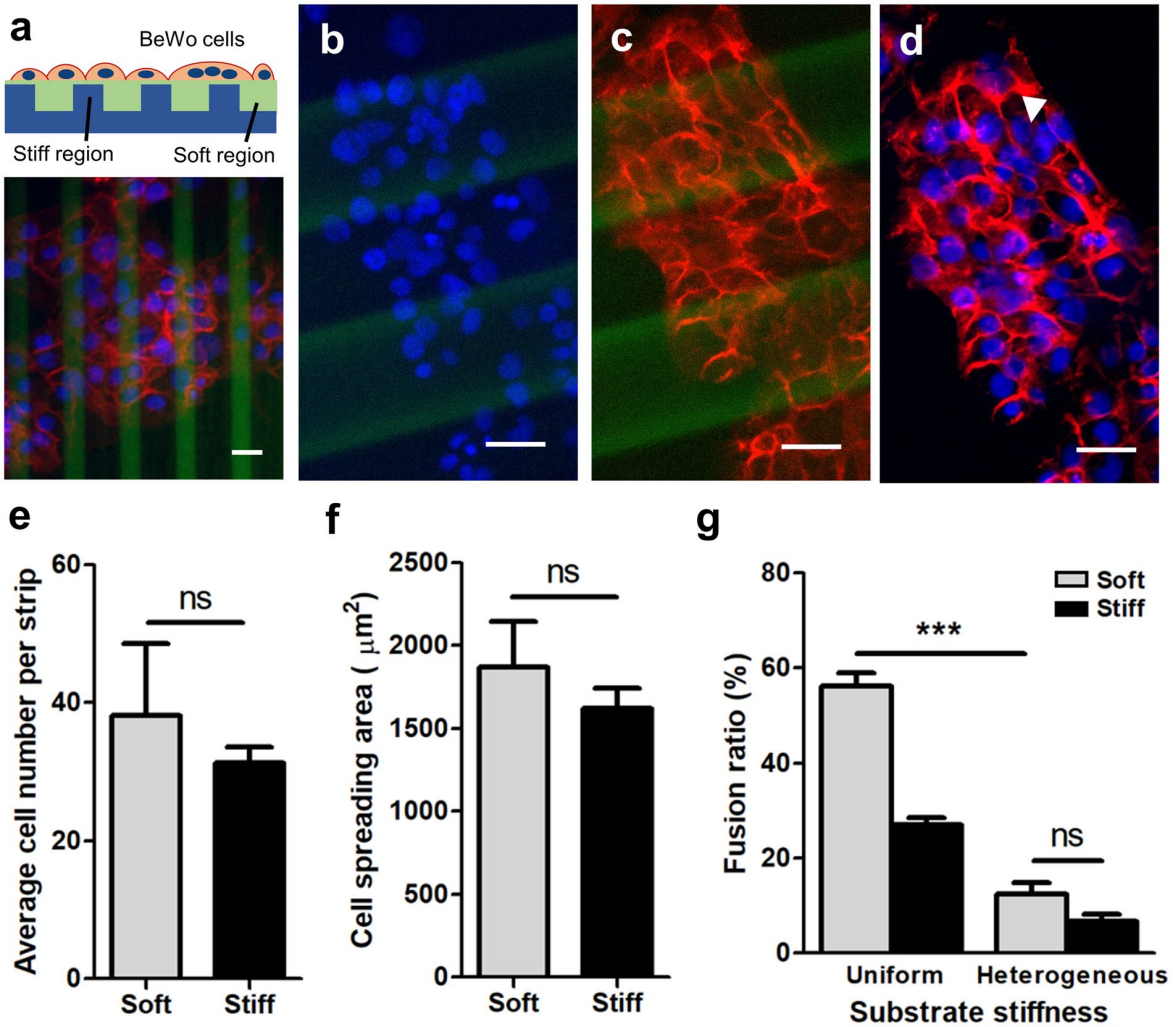
Effects of heterogeneous substrate stiffness on BeWo cell fusion. (**a**) Schematic of the mechanically patterned composite hydrogel for cell culture (top) and the representative figures of BeWo cells cultured after 48-hour forskolin induction. (**b–d**) Representative images of (**b**) nuclei, (**c**) E-cadherin, and (**d**) merged on heterogeneously stiff substrates. Red: E-cadherin; blue: DAPI; green: fluorescein. Scale bar is 50 μm. (**e**,**f**) No significant difference was observed regarding (**e**) average cell number per strip and (**f**) cell spreading area. (**g**) BeWo cell fusion ratio was greatly suppressed when cultured on heterogeneous substrate with stiff “hot spots”. Data reported as mean ± standard deviation for n = 3 independent experiments; ***p < 0.001, by Student’s *t*-test.

“Stripes” of increased stiffness have previously been microfabricated into polyacrylamide hydrogel substrates to guide myotube fusion and striation^56^, and direct stem cell differentiation^57^. Using a similar technique, a composite hydrogel substrate consisting of parallel soft (1.3 kPa) and stiff (17.4 kPa) strips (Fig. 6a–d; labeled green with fluorescein in the polymer backbone^58^) of varying thickness was fabricated to simultaneously provide colonies of cells with low- and high- stiffness cues. No differences were observed in cell attachment, spreading, or fusion based on variations in stripe width or spacing (25–100 µm), and hence all striped patterns were pooled as “heterogenous” substrates. After 48 hours, BeWo cells demonstrated well-spread morphologies on both soft and stiff regions of the substrate, similar to those observed on homogenously stiff substrates (Fig. 6f), suggesting that regions of high stiffness provides a dominant microenvironmental cue over softer regions. To quantify fusion, we first confirmed that the numbers of cells adhering to both soft and stiff regions were statistically similar (Fig. 6e), and then characterized the fusion ratio for those cells on soft and stiff regions, within all larger colonies that spanned at least two hydrogel strips. Fusion on both soft and stiff regions was always significantly lower on the patterned substrates, than on uniformly soft substrates (Fig. 6g). Taken together, these findings suggest that substrates with stiffened ‘hot spots’ associated with focal stiffening processes such as fibrosis affects fusion of trophoblasts on adjacent soft regions with normal tissue stiffness. Speculatively, these observations suggest that fusion can be influenced at early stages of disease progression, through a focal stiffening mechanism.

## Discussion

Although substrate stiffness has been established as a critical regulator of several developmental processes, the importance of disease-specific extracellular mechanics has not previously been established for trophoblast fusion and function. In this work, we demonstrate that substrate stiffness that mimics placental tissue in normal and disease states regulates placental trophoblast morphology, fusion, and secretion of proteins. We develop these findings in a model cell line that is widely used to study fusion specifically, and also verify our studies with primary trophoblasts isolated from placenta at term. Our experiments confirm that the fusion process is regulated by substrate mechanics through myosin-II activity, cannot be predicted by local cell spread area, and that simple patterns of extracellular stiffness may have dominant effects over adjacent mechanically normal tissue. Taken together, these results demonstrate that in the context of normal and diseased placental tissue characteristics, mechanobiological regulation of trophoblast fusion likely plays a critical role in cell-mediated disease progression.

The precise mechanism for mechanobiological regulation of trophoblast fusion remains an open question. The complex nature of reciprocal interactions along the matrix-integrin-cytoskeleton axis in mechanotransduction^37,59,60^ remains to be explored for trophoblast fusion specifically, and other groups have already identified biophysical features such as cytoskeletal disruption, actin dynamics and membrane flexibility to be necessary for trophoblasts to become fusion-competent^61^. Fusion may also be regulated by the mechanical balance between matrix-mediated traction forces, internal tension generated by the actin cytoskeleton, and intercellular tugging forces^62^ that may each contribute to a fusion event. Moreover, the 3D patterned presentation of adhesive ligands may also affect mechanobiological function^63^. Finally, these fundamental mechanisms are likely co-regulated by other disease-related features, including soluble factors^17^, matrix composition^64^, membrane proteins such as syncytin^22^ and more complex mechanical features of the substrate such as non-linear elasticity and viscoplasticity^65^; suggesting the need for highly combinatorial studies of factors affecting this process.

More broadly however, this work demonstrating that trophoblast fusion and function are mechanically sensitive highlights the importance of extracellular tissue engineering strategies to better understand and rationally drive development of tissue engineered models for both fundamental studies and applied research in drug development for placental disease. Knowledge of mechanically-mediated mechanisms for fusion could hence be further leveraged to create a well-fused placental barrier, for the design of next-generation drug screening strategies such as placenta-on-a-chip *in vitro* culture models^66^. Furthermore, establishing the mechanical nature of this fundamental process will allow us to identify novel mechanistic regulators of fusion, ultimately suggesting novel directions for the development of pregnancy-related therapeutic strategies.

## Methods

Ethical approval for the study obtained from institutional review board at the CHUM St-Luc Hospital (Montreal, QC, Canada). All experimental methods were performed in accordance with relevant guidelines and regulations, including McGill Biosafety regulations. Human samples were obtained from term delivery after written informed consent was obtained from all the patients involved in the study (CER-16–427).

Unless otherwise stated, all cell culture materials and supplies were purchased from Fisher Scientific (Ottawa, ON), and chemicals from Sigma Aldrich (Oakville, ON).

### Polyacrylamide hydrogel fabrication and surface treatment

Polyacrylamide hydrogels of different stiffness were fabricated on 12 mm glass coverslips. Four rigidities were obtained by altering the monomer/crosslinker ratios reported previously^35^, including sub-physiological (G = 0.1 kPa), normal (G = 1.3 kPa), pathological (G = 7 kPa) and extreme pathological (G = 17.4 kPa). The final concentration of acrylamide/bis-acrylamide for gels with desired stiffness were listed in Table 1. Briefly, glass coverslips were silanized with 0.4% (v/v) solution of 3-(trimethyoxysilyl) propyl methacrylate in acetone for 5 minutes. The coverslips were then rinsed with acetone twice and air-dried. A 45 μL pre-polymer solution containing monomer acrylamide (40% w/v, Bio-Rad Laboratories, Hercules, CA), crosslinker N,N-methylene-bis-acrylamide (BIS, 2% w/v, Bio-Rad) stock solutions, photo-initiator ammonium persulfate (Bio-Rad) and catalyst N-N-N-N-tetramethylethylenediamine were sandwiched between the silanized 12 mm glass coverslip and a hydrophobic glass slide. The gelation time for hydrogels with different stiffness varied from 5 to 20 minutes. Once polymerized, the gels were peeled from the glass slide and transferred to a 12-well plate. They were then washed with phosphate buffered saline (PBS) overnight and sterilized under a germicidal UV lamp for 45 minutes.

**Table 1:** Hydrogel composition and rigidity.

Stiffness (kPa)	0.1	1.3	7	17.4
40% Acrylamide (µl)	75	100	200	400
2% Bisacrylamide (µl)	26	150	240	480
PBS (µl)	799	650	460	0
TEMED (µl)	1.5	1.5	1.5	1.5
1% APS (µl)	100	100	100	120
TOTAL (µl)	1001.5	1001.5	1001.5	1001.5

Composite polyacrylamide hydrogels with heterogeneous stiffness patterns were fabricated using a two-step hydrogel polymerization process on a microfabricated template with groove features. The template was prepared using standard SU-8 lithography and transferred to an epoxy mold. Stiff polyacrylamide hydrogels (17.4 kPa) were first cast on the microfabricated template and peeled away. A second soft polyacrylamide hydrogel layer (1.3 kPa) was then cast into the groove spaces and sandwiched under a glass slide. 1 μL of 10% w/v fluorescein o-methacrylate (Sigma-Aldrich, 568864) in dimethyl sulfoxide (DMSO) was added to stiff hydrogel pre-polymer solution for the synthesis of fluorescent hydrogel to identify stiff regions on composite hydrogel substrates.

To functionalize the polyacrylamide surface and facilitate cell adhesion, Type I bovine collagen (Advanced Biomatrix) was coated on the gel surface, using the photoactivatable bifunctional crosslinker N-sulfosuccinimidyl-6-[4′-azido-2′-nitrophenylamino] hexanoate (sulfo-SANPAH, ProteoChem). Briefly, gels were immersed in a 0.05 mg/mL solution of sulfo-SANPAH in PBS and placed under UV lamp for 4 minutes until the solution turned from orange to a pale-yellow color. This process was repeated twice. After thoroughly rinsing the gels in PBS, the hydrogels were incubated in 0.05 mg/mL collagen I overnight at 4 °C, and rinsed twice in PBS prior to cell culture. Glass coverslips used as control experiments were also incubated in 0.05 mg/mL collagen I solution overnight prior to use.

### Mechanical characterization of polyacrylamide hydrogels

The stiffness of the polyacrylamide hydrogels was characterized using a parallel plate, strain-controlled rheometer (Anton Paar MCR 302). Following previously-established procedures in our lab for polyacrylamide gel characterization^58^, pre-polymer solution was sandwiched between silanized glass coverslips and polymerized to produce hydrogel disks with 1 mm thickness, which were hydrated in PBS overnight. A strain amplitude within the linear viscoelastic range (10%) was applied over a frequency range of 0.3–300 rad/s was applied on the gels. Excess PBS was wicked from the top and bottom of the hydrogel disks and attached to the rheometer plates with double-sided tape. The storage modulus was measured at 10% strain from 0.001 to 10 Hz, and verified to be linear elastic with a strain amplitude sweep from 1 to 10% strain at 1 Hz oscillatory frequency^58^.

### Cell isolation and culture

BeWo cells (CCL-98 clone), from American Type Culture Collection (ATCC; Rockville, MD), were cultured in Dulbecco’s Modified Eagle Medium (DMEM)/F-12 with phenol red and supplemented with 10% fetal bovine serum (FBS; Hyclone, Tempe, AZ) and 1% penicillin-streptomycin (Hyclone). Only BeWo cells with passage number lower than 20 were used in these experiments. Trypsinized cells were seeded at densities of 1 × 10^4^ cells/mL. 24 hours after seeding cells on collagen I-coated hydrogels, cells were treated with 20 μM forskolin (Sigma-Aldrich, F6886) for 48 hours. The forskolin-containing medium was changed daily during the experiments. For cell morphology analysis, BeWo cells were exposed to mitomycin C (10 mg/ml) for 2 hours to inhibit proliferation and washed three times with culture media prior to plating. To selectively inhibit nonmuscle myosin II, culture medium was supplemented with 50 µM Blebbistatin.

Human primary vCTBs, were obtained from term placentas from vaginal delivery of uncomplicated pregnancies, and were isolated using previously used methods^67^. Briefly, placental tissue was consecutively digested in trypsin and DNase; after which each of the supernatants were collected, pooled, and separated via a Percoll gradient. Mononuclear villous trophoblasts were immunopurified by negative purification, coupling an anti-HLA-ABC antibody (Biolegends) followed by anti-mouse secondary antibody-coupled magnetic beads (Miltenyi Biotec, Santa Barbara, CA), and sorted using an autoMACS (Miltenyi). All vCTB preparations were at least 95% pure after cell sorting^68,69^. vCTBs were mixed in a 1:10 solution of DMSO in FBS cryoprotectant, frozen overnight at −80 °C, and stored in liquid nitrogen^67^. Primary vCTBs were plated at 1.6 × 10^6^ cells/mL, allowed to attach for 4 hours, and gently rinsed with warm media to wash away mononuclear syncytial fragments^69,70^. vCTBs were maintained at 37 °C in high glucose DMEM, supplemented with 10% FBS and 1% penicillin/streptomycin, in hyperoxic conditions (21% O_2_). Primary cells from each donor were seeded on each of the differentially stiff hydrogels (and plastic control surfaces). Cells from different donors were not pooled to avoid intragroup variability.

### Immunostaining

Staining techniques for fused BeWo cells on patterned and hydrogel substrates were previously established in our lab^67^. Following our previous protocols, cells were fixed in 4% (w/v) paraformaldehyde in PBS for 15 minutes at room temperature; and embedded in a thin layer of porous polyacrylamide hydrogel (100 Pa stiffness) to prevent detachment of the weakly-adhered syncytial patches after fusion. This process was previously confirmed to have no effect on the immunostaining results^67^. Substrates were washed twice with PBS, permeabilized in 0.1% (v/v) triton X-100 in PBS for 15 minutes, and washed twice with PBS again. Samples were blocked in 2.5% (v/v) goat serum in PBS for 2 hours at room temperature to prevent non-specific binding. As per previous protocols for indirect staining^67^, cells were incubated with anti-E-cadherin antibody (1:200, Abcam, ab1416; overnight, 4 °C) or anti-β-hCG antibody (1:500, Thermo Fisher, #14-6508-82; overnight, 4 °C) in goat serum. For secondary staining, cells were washed twice with PBS and incubated with goat anti-mouse IgG H&L (Alexa Fluor. 488) antibody (1:1000, Abcam, ab150113; 3 hours). Directly stained cells were incubated with 1:200 DAPI (Invitrogen) and FITC-phalloidin (Invitrogen) in goat serum solution (2 hours, room temperature), and washed thoroughly with PBS^67^.

### Image collection and analysis

Imaging was performed on an inverted fluorescent Olympus microscope (Olympus, IX73) outfitted with an sCMOS Flash 4.0 Camera and Metamorph software (version 7.8.13.0). All images were taken at randomly selected locations at 20X magnification. Subsequently, the obtained images (f-actin, E-cadherin, or hCG with DAPI) were merged and analyzed using ImageJ (NIH). Cell morphology was characterized based on fluorescent images obtained after 24-hours of culture with mitomycin C inhibition to prevent proliferation. The ratio of cells expressing hCG were analyzed based on the co-localization of hCG and DAPI-labelled nuclei signals in merged immunofluorescence figures. Fusion was assessed in BeWo cells after 48-hour forskolin induction and vCTBs after 72-hours of primary culture based on immunofluorescent localization of E-cadherin and DAPI-labelled nuclei. Any cluster of 3 or more nuclei enclosed within an E-cadherin boundary was considered a syncytium, consistent with established characterization guidelines^31^. The total number of nuclei (T), syncytium number (S) and total nuclei number in fused syncytium (F) were counted. The ratio of cell fusion was calculated using the following standard equation: (F − S + 1)/T × 100.

### Released β-hCG analysis

Released β-hCG was quantified following a previous protocol for a similar experiment^67^. Briefly, conditioned cell culture media was collected after 48 hours of forskolin induction, centrifuged, separated and stored at −20 °C. β-hCG concentration was measured by enzyme-linked immunosorbent assay (ELISA) against the relevant antibody (DRG beta-hCG ELISA (EIA-1911); IBL International, Toronto, ON, Canada), by incubating aliquots of the collected media in enzyme conjugate-coated microtiter wells (15 minutes, room temperature). A microtiter plate reader was used to determine absorbance of each well at 450 nm^67^.

### Statistical analysis

Comparative data analysis and standard deviations of fusion ratios were based on results obtained from 3 independent experiments conducted on different days. Statistical significance of fusion ratios, cell spreading area and β-hCG expression level amongst cells cultured on substrates with different stiffness was analyzed using Student’s *t*-test or a two-tailed, one-way ANOVA with a Holm-Sidak post-hoc pairwise comparison test. All statistical analyses were conducted in SigmaStat 3.5 (Systat Software Inc., San Jose, CA).

## Data Availability

The datasets generated during and/or analyzed during the current study are available from the corresponding author on reasonable request.
